# Risk factor clusters for non-communicable diseases in adolescents in Eastern Cape, South Africa

**DOI:** 10.4102/hsag.v30i0.2855

**Published:** 2025-01-15

**Authors:** Avela Mathe, Maya M. van Gent, Rudolph L. van Niekerk

**Affiliations:** 1 Department of Human Movement Science, Faculty of Health Sciences, University of Fort Hare, Alice, South Africa; 2 Department of Psychology, Faculty of Social Sciences and Humanities, University of Fort Hare, East London, South Africa

**Keywords:** adolescents, metabolic risk factors, NCDs, perceived stress, South Africa

## Abstract

**Background:**

The increasing prevalence of non-communicable diseases (NCDs) among adolescents in South Africa is a growing public health concern.

**Aim:**

To examine the clustering of NCD risk factors, with a focus on gender, socio-economic status (SES) and perceived stress among adolescents.

**Setting:**

The study involved adolescents from eight schools in Buffalo City Metropolitan Municipality and Amatole district, Eastern Cape.

**Methods:**

A stratified random sample of 266 adolescents (aged 12 years–18 years) was assessed for anthropometric, physiological, and perceived stress measures. Data included body mass index (BMI), blood pressure (BP), cholesterol, glucose levels, and perceived stress (via the Perceived Stress Scale). Analysis involved descriptive statistics, Chi-square tests, *t*-tests, and k-means clustering.

**Results:**

Females (*n* = 155) showed a higher prevalence of NCD risk factors, with 80.2% classified as overweight or obese compared to 19.8% of males, and 77.2% had elevated cholesterol versus 22.8% of males. Perceived stress was higher in affluent schools (59.8%) despite fewer metabolic risks. K-means analysis identified four health profiles with significant SES and health differences (*p* < 0.001). Cluster 1 (high SES) showed elevated BP, BMI, and stress, while Clusters 2–4 (low SES) varied in risks, with Cluster 4 showing the highest BP and metabolic risks despite low stress.

**Conclusion:**

These results highlight unique health profiles and risk factors across socio- economic contexts, with female adolescents from lower socio-economic backgrounds facing greater metabolic risks.

**Contribution:**

This Study provides original insights into the interplay between gender, SES and perceived stress in shaping NCD risk among South African adolescents.

## Introduction

Non-communicable diseases (NCDs) are a leading cause of premature death worldwide, accounting for approximately 70.0% of adult deaths globally, and 57.8% in South Africa (Akseer et al. 2020; WHO 2019). The burden of NCDs is rising rapidly, particularly in low- and middle-income countries where healthcare systems are often overstretched. In Africa, NCD-related mortality is expected to increase by over 27% in the next decade (Gouda et al. 2019; Masocha, Monyeki & Czyż 2020). Alarmingly, NCDs contributed to 11% of deaths among children and adolescents in 2018 (WHO 2020). Many of the risk factors for NCDs, such as obesity, hypertension and diabetes, begin to manifest in adolescence and, if left unaddressed, can worsen into adulthood, significantly increasing the risk of early mortality (Gensthaler et al. 2022). Early identification and intervention during adolescence are, therefore, critical to mitigating the long-term impact of these diseases.

In South Africa, the rising risk of NCDs among adolescents is a growing public health concern. One of the most pressing issues is obesity, with 27% of girls and 9% of boys aged 5–18 years classified as overweight or obese (Wrottesley et al. 2020). These figures are particularly concerning in rural and socio-economically disadvantaged areas, where poor nutrition and physical inactivity exacerbate NCD risks. Adolescents in rural areas are especially vulnerable to NCD risk factors because of food insecurity and limited access to healthcare services (Draper et al. 2019; Otitoola, Oldewage-Theron & Egal 2021). Another significant NCD risk factor is hypertension, which is increasingly prevalent among South African adolescents, with reported rates ranging from 1.0% to 25.9% (Bhimma et al. 2018; Nqweniso et al. 2020; Jones et al. 2020). Hypertension is more common in boys (21.62%) than girls (14.29%), particularly in rural areas of the Eastern Cape (Hinton et al. 2020). Elevated blood pressure (BP) is often linked to lifestyle factors, such as reduced physical activity and sedentary behaviour (Nkeh-Chungag et al. 2015).

High cholesterol and type 1 diabetes further compound the burden of NCDs among South African adolescents. In Cape Town, 28.6% of adolescents have been diagnosed with dyslipidaemia (Dookhony, Lombard & Zöllner 2022). While comprehensive data on diabetes in this age group is limited, evidence suggests that the prevalence is likely to rise, adding to the NCD burden (Divers et al. 2020). Addressing these NCD risks is essential for improving long-term health outcomes and reducing future healthcare costs. This study, therefore, focusses on adolescents in the Eastern Cape, one of the poorest provinces in South Africa. It aims to explore the relationship between socio-economic status (SES), psychosocial stress, and NCD risk. By identifying key risk factors, this research will help to inform the development of targeted interventions designed to reduce NCD prevalence and improve health outcomes in vulnerable adolescent populations.

Despite the growing global evidence of increasing NCD rates among adolescents, research specific to the South African context remains limited, particularly in rural or socio-economically disadvantaged areas. Although several studies have documented high rates of obesity, hypertension, and high cholesterol among South African adolescents, few have explored the influence of psychosocial factors, such as perceived stress, on these risk factors (Gooding et al. 2020; Nqweniso et al. 2020). Moreover, there is little research on how socio-economic disparities affect NCD risk in rural versus urban schools. Much of the existing research focusses on urban centres or more affluent regions, leaving rural areas under-represented (Rosengren et al. 2019). This study seeks to address this gap by examining how NCD risk factors interact with perceived stress among adolescents in lower-quintile schools in the Eastern Cape province. By focussing on gender, school quintiles and perceived stress, this research provides original insights into the socio-environmental determinants of NCD risk, offering new perspectives on how to mitigate these risks in under-resourced settings.

Chronic stress has been closely linked to metabolic dysregulation, making adolescents who experience higher levels of stress more susceptible to NCD development (Lindholdt et al. 2022; Reilly & Kelly 2011). Adolescents from lower socio-economic backgrounds are particularly prone to chronic stress because of factors such as poverty, limited resources and social instability, all of which can exacerbate their physiological vulnerability to NCDs (Stormacq et al. 2019).

In South Africa, particularly in the Eastern Cape province, there is a significant lack of research examining the relationship between perceived stress and NCD risk among adolescents. Given the socio-economic challenges faced by many adolescents in this region, challenges are compounded by poverty and a lack of resources in the school setting (Ella, Shehab & Ismail 2011; Francis & Webster 2019; Khumalo 2023). Many communities are affected by unemployment and financial instability, which contribute to food insecurity and malnutrition, negatively affecting adolescents’ well-being and resulting in a lower quality of learning (Kanjere & Choenyane 2021; Pillay et al. 2023). It is crucial to understand how these stressors interact with physiological risk factors to increase vulnerability to NCDs. Therefore, this study aims to compare adolescents aged 12–18 years who are at risk for NCDs with those who are not, taking into account variables such as gender, school quintiles and perceived stress.

## Research methods and design

### Research setting and design

This study is part of a broader research project titled ‘An intervention to address the physical, physiological, and psychological risk factors linked to non-communicable diseases among adolescents in the Eastern Cape, South Africa’. A quantitative research approach was employed, focussing on schools within the Buffalo City Metropolitan Municipality and the Amatole district in the Eastern Cape province. The Eastern Cape, South Africa’s second-largest province by land area, has a population of approximately 6.6 million, making it the third most populous province in the country.

### Study population

A stratified random sampling was used to target adolescents aged 12–18 years from eight randomly selected schools in two Eastern Cape districts: Amatole district and Buffalo City Metropolitan Municipality. A computer-based randomisation tool was used in Microsoft Excel to select four schools from each district to include schools from quintiles one, two and three (no-fee paying schools), representing socio-economically disadvantaged groups, and schools from quintiles four and five (fee-paying schools), representing more affluent socio-economic groups. The total population of South African adolescents is approximately 9 950 100, with 2 473 140 residing in the Eastern Cape province (Stats SA 2018). The final sample consisted of 266 adolescents, comprising 41.7% males (*n* = 111) and 58.3% females (*n* = 155), which resulted in a 5.89% margin of error.

### Data collection

Height was measured in centimetres using a stadiometer, with participants standing barefoot. Weight was measured in kilograms using a calibrated scale, with participants wearing minimal clothing to ensure accuracy. Body mass index (BMI) was calculated as weight divided by height squared (kg/m^2^). Waist-to-hip ratio was determined using girth measurements, including arm, hip and waist girth, with participants standing in a relaxed position. Blood pressure measurements were taken using a CONTEC08A/C digital BP monitor, with appropriately sized arm cuffs fitted on the left arm. Measurements were taken after participants were seated for 5 min. Random blood glucose and cholesterol levels were assessed through finger-prick tests administered by a professional nurse using the Accutrend Plus Cobas machine. Random blood glucose was measured in millimoles per litre (mmol/L) and cholesterol levels were recorded in mmol/L. Perceived stress was measured using the Perceived Stress Scale (PSS), a widely validated self-report instrument designed to assess stress levels in adolescents and adults (Cohen, Kamarck & Mermelstein 1983). The PSS consists of 10 items rated on a five-point Likert scale, with higher scores indicating greater perceived stress. This scale has been validated for use among South African adolescents (Makhubela 2022) and demonstrated good internal consistency with a Cronbach’s alpha coefficient of 0.632.

Body mass index classifications were based on the following categories: underweight (< 18.5 kg/m^2^), normal weight (18.5 kg/m^2^ – 24.9 kg/m^2^), overweight (25.0 kg/m^2^ –29.9 kg/m^2^) and obese (> 30.0 kg/m^2^) (Draper et al. 2019). Blood pressure was classified according to the following ranges: normal (≤ 120/80 mmHg) and at risk (≥ 121/81 mmHg) (Demir, Bilgin & Caner 2020; WHO 2020). For blood glucose, levels exceeding 11.1 mmol/L indicated diabetes among adolescents aged 13–19 years (WHO 2019). Cholesterol levels were classified as normal (< 5.17 mmol/L), borderline high (5.17 mmol/L – 6.18 mmol/L) and high (> 6.21 mmol/L) (Hassan, El Sayed & El-aasar 2020).

### Data analysis

Adolescents presenting with one or more metabolic risk factors (overweight or obesity, elevated systolic BP, diastolic BP, elevated random blood glucose, elevated blood cholesterol) were classified as at risk for NCDs. Metabolic risk factors were used as proxies for NCD risk in this study. The data were analysed using IBM Statistical Package for the Social Sciences (SPSS) Statistics (Version 27.0, IBM Corp., 2021). Descriptive statistics were calculated as means ± standard deviations for continuous variables. Chi-square analyses were used to examine associations between categorical variables, such as gender, school quintiles and NCD risk. Independent samples *t*-tests were employed to compare at-risk and not-at-risk adolescents across various demographic and physiological variables. In addition, a *k*-means cluster analysis was done once all continuous variables were standardised (*z*-scores) to ensure equal weighting. The optimal number of clusters was determined based on the interpretability of clusters and the percentage of variance explained (using the ANOVA [analysis of variance] *F*-statistic as a descriptive tool). A three-cluster solution was initially tested, but the four-cluster solution provided more interpretable and distinct profiles without significantly increasing within-cluster variance. To determine the extent to which each variable differentiated the clusters, an ANOVA was conducted on each variable with the cluster membership as the grouping factor. A significance level of *p* < 0.05 was used for all statistical tests, and 95% confidence intervals were reported.

### Ethical considerations

This study was part of a larger study, titled: ‘An intervention to address the physical, physiological, and psychological risk factors associated with non-communicable diseases among adolescents in the Eastern Cape, South Africa’. Ethical clearance (Ref#2022=02=09=MatheA) was granted by the Health Research Ethics Committee (REC-100118-054) at the University of Fort Hare. The Eastern Cape Department of Basic Education and the principals of the participating schools provided consent for the study. Written informed consent was obtained from the parents or legal guardians of the adolescents, and all learners signed assent form prior to their participation in the study. Each participant was assigned a unique identification number to ensure anonymity and privacy. Participants received questionnaires with their codes only and not their names, with all data recorded and managed using this identifier.

## Results

Table 1 provides a demographic overview of the study participants. The sample included 266 adolescents, of whom *n* = 111 (41.7%) were male and *n* = 155 (58.3%) were female. Participants were drawn from both rural and urban areas, with 58.3% attending schools in rural districts and 65.4% from quintiles 1–3 schools (disadvantaged schools). A statistically significant association was found between gender and age (*p* = 0.007), with the majority of females being younger (70% of 14-year-olds were female), while males dominated the older age groups (57.4% of 16-year-olds were male). No significant differences were observed in terms of district (*p* = 0.915) or SES (*p* = 0.100) between genders.

**TABLE 1:** Demographic and health characteristics by gender, district, socio-economic status, age and non-communicable disease risk factors among adolescents.

Demographics	Demographics subgroups	Gender				Total		Sign	Effect size
		Male		Female					
		*n*	%	*n*	%	*n*	%	*p*	Phi
District	U	60	41.1	86	58.9	146	41.7	0.915	0.014
	R	51	42.5	69	57.5	120	58.3	-	-
SES	Q1–3	73	42.0	101	58.0	174	65.4	0.100	0.006
	Q4–5	38	41.3	54	58.7	92	34.6	-	-
Age	13	9	32.1	19	67.9	28	10.5	0.007*	0.258
	14	21	30.0	49	70.0	70	26.3	-	-
	15	30	36.6	52	63.4	82	30.8	-	-
	16	31	57.4	23	42.6	54	20.3	-	-
	17	15	60.0	10	40.0	25	9.4	-	-
	18	4	66.7	2	33.3	6	2.3	-	-
Cum	No	73	68.2	53	35.3	126	68.2	0.000*	0.333
	1 f	28	26.2	70	46.7	98	28.6	-	-
	2 f	5	25.0	47	16.7	30	4.7	-	-
	3 f	1	0.9	2	1.3	3	0.9	-	-
BP	No risk	99	41.1	142	58.9	241	90.6	0.002*	0.041
	Risk	12	48.0	13	52.0	25	9.4	-	-
Chol	No risk	94	47.0	106	53.0	200	77.8	0.002*	0.001
	Risk	13	22.8	44	77.2	57	22.2	-	-
Glu	No risk	111	41.7	155	58.3	266	-	-	-
BMI	No risk	93	53.1	82	46.9	175	65.8	0.000*	0.321
	Risk	18	19.8	73	80.2	83	34.5	-	-
PS	Low	66	48.9	69	51.1	135	52.7	0.032*	0.164
	Mod	38	35.5	69	64.5	107	41.8	-	-
	High	3	21.4	11	78.6	14	5.5	-	-

SES, socio-economic status; F, risk factor; cum, accumulation of metabolic risk factors; BP, blood pressure; chol, cholesterol; Glu, glucose; BMI, body mass index; PS, perceived stress; U, urban; R, rural.

* , Significance = *p* < 0.05.

There was a significant association between gender and the number of NCD risk factors (*p* = 0.000). Among male participants, 68.2% had no risk factors, while female participants showed a higher accumulation of risk factors, more specifically 46.7% of females had at least one risk factor. Furthermore, significant differences were found in several metabolic risk factors, which include BP = 0.002. Females were significantly more likely to have elevated cholesterol levels, with 77.2% having elevated cholesterol compared to 22.8% of males (*p* = 0.001). Also, a significantly larger proportion of females, 80.2%, were classified as overweight or obese, compared to 19.8% of males (*p* = 0.000).

Table 2 focusses on perceived stress levels across demographic variables and metabolic risk factors. A significant association was found between SES and perceived stress (*p* = 0.000). Participants from quintiles 1–3 (disadvantaged schools) were more likely to report low stress levels (70.1%), while those from quintiles 4–5 (privileged schools) were more likely to report high stress (59.8%). This suggests that students from more affluent backgrounds may face higher psychological stress despite their lower risk of metabolic factors. There was also a significant association between gender and perceived stress (*p* = 0.032). Among female participants, 78.6% reported high stress levels, while only 21.4% of males reported similar levels of stress. Moderate stress was also more prevalent among females (64.5%) compared to males (35.5%). These results point to a gender difference in stress experience, with females reporting significantly higher stress levels than males. However, no significant associations were found between perceived stress and other demographic variables such as age (*p* = 0.760), district (*p* = 0.486), or cumulative risk factors (*p* = 0.657).

**TABLE 2:** Demographic characteristics and associations between perceived stress levels and non-communicable disease risk factors among adolescents.

Demographics	Demographic subgroups	Stress				Total		Sign	Effect size
		Low		High					
		*n*	%	*n*	%	*n*	%	*p*	Phi
District	R	75	62.5	45	37.5	120	45.1	0.486	0.243
	U	84	57.5	62	42.5	146	54.9	-	-
SES	Q1–3	122	70.1	52	29.9	174	65.4	0.000*	0.290
	Q4–5	37	40.2	55	59.8	92	34.6	-	-
Age	13	18	64.3	10	35.7	28	10.5	0.760	0.107
	14	42	60.0	28	40.0	70	26.3	-	-
	15	52	63.4	30	36.6	82	30.8	-	-
	16	28	51.9	26	48.1	54	20.3	-	-
	17	14	56.0	11	44.0	25	9.4	-	-
	18	4	66.7	2	33.37	6	2.1	-	-
Cum	No	78	61.9	48	38.1	1	49.0	0.657	0.080
	1 f	58	59.2	40	40.8	126	38.1	-	-
	2 f	16	53.3	14	46.7	98	11.7	-	-
	3 f	1	33.3	2	66.7	30	1.2	-	-
BP	No risk	143	59.3	98	40.7	241	90.6	0.812	0.028
	Risk	16	64.0	9	36.0	25	9.4	-	-
Chol	No risk	120	60.0	80	40.0	200	77.8	0.894	0.018
	Risk	33	57.9	24	42.1	57	22.2	-	-
Glu	No risk	159	59.8	107	40.2	266	-	-	-
BMI	No risk	109	62.3	66	37.7	175	65.8	0.305	0.071
	Risk	50	54.9	41	45.1	91	34.2	-	-

SES, socio-economic status; F, risk factor; cum, accumulation of metabolic risk factors; BP, blood pressure; chol, cholesterol; Glu, glucose; BMI, body mass index; PS, perceived stress; U, urban; R, rural.

* , Significance = *p* < 0.05.

Table 3 presents the correlation analysis between physiological risk factors and perceived stress. The analysis revealed mostly weak but statistically significant positive correlation between BMI and perceived stress (*r* = 0.131; *p* = 0.032) and between systolic BP and BMI (*r* = 0.199; *p* = 0.001). A significant weak positive correlation (*r* = 0.185; *p* = 0.003) was found between higher blood glucose levels and systolic BP. Age was also positively correlated with systolic BP (*r* = 0.192; *p* = 0.002) and diastolic BP (*r* = 0.138; *p* = 0.024), indicating that older adolescents are more likely to experience elevated BP levels. There were no significant correlations between perceived stress and other physiological risk factors such as blood glucose or cholesterol levels. These results suggest that while BMI and systolic BP are related to perceived stress, other metabolic risk factors may not be directly influenced by stress levels.

**TABLE 3:** Correlations between age, perceived stress, blood pressure, blood glucose, cholesterol, and body mass index among adolescents.

Variables	Value	Age	Perceived stress	Systolic	Diastolic	Blood glucose	Blood cholesterol	BMI
Age	*r* *p*	1.000 -	0.056 0.364	0.192 0.002*	0.138 0.024*	−0.032 0.609	−0.068 0.027*	0.082 0.181
Perceived stress	*r* *p*	0.056 0.364	1.000 -	−0.042 0.498	0.021 0.732	0.023 0.708	−0.060 0.339	0.131 0.032*
Systolic	*r* *p*	0.192 0.002*	0.042 0.498	1.000 -	0.687 0.000*	0.185 0.003*	0.074 0.236	0.199 0.001*
Diastolic	*r* *p*	0.138 0.024*	0.021 0.732	0.687 0.000*	1.000 -	0.144 0.065	0.069 0.271	0.132 0.031*
Blood glucose	*r* *p*	−0.032 0.609	0.023 0.708	0.185 0.003*	0.114 0.065	1.000 -	0.128 0.038*	0.046 0.454
Blood cholesterol	*r* *p*	−0.068 0.274	−0.060 0.339	0.074 0.236	0.069 0.271	0.128 0.038*	1.000 -	0.015 0.816
BMI	*r* *p*	0.082 0.181	0.131 0.032*	0.199 0.001*	0.132 0.031*	0.046 0.454	0.015 0.816	1.000 -

BMI, body mass index.

*r* = correlation; *p* = *, significance (*p* < 0.05).

Table 4 represents the results from the *k*-means cluster analysis. Before the cluster analysis was done, all continuous variables were standardised (*z*-scores) to ensure equal weighting. The results for the cluster analysis identified four distinct clusters, each exhibiting unique socio-economic and health characteristics, as revealed by the final cluster centres and confirmed by the ANOVA results presented in Table 4.

**TABLE 4:** Final cluster centres and analysis of variance.

Variables	Clusters				ANOVA	
	1	2	3	4	*F* ***	Sig.
Gender*	2	2	2	2	7.916	< 0.001
Socio-economic status (Quintiles)**	2	1	1	1	5.658	< 0.001
*Z*-score: Systolic BP	0.32817	0.45062	−0.86262	0.69292	73.838	< 0.001
*Z*-score: Diastolic BP	0.29325	0.57319	−0.86521	0.59512	74.313	< 0.001
*Z*-score: Blood glucose	0.12116	−0.53832	−0.18295	0.80106	27.776	< 0.001
*Z*-score: Blood cholesterol	0.27271	−0.64904	0.05652	0.44980	17.165	< 0.001
*Z*-score: Perceived stress	1.10722	0.28887	−0.16760	−0.55247	28.534	< 0.001
*Z*-score: BMI	1.73270	−0.26529	−0.37002	−0.04034	66.716	< 0.001

BP, blood pressure; BMI, body mass index; ANOVA, analysis of variance; Sig., significance.

* , Gender (1 = male; 2 = female);

** , socio-economic status (1 = less affluent quintile schools; 2 = more affluent quintile schools);

*** , the F tests should be used only for descriptive purposes because the clusters have been chosen to maximise the differences among cases in different clusters. The observed significance levels are not corrected for this and thus cannot be interpreted as tests of the hypothesis that the cluster means are equal.

The cluster analysis results revealed distinct health and socio-demographic characteristics across four identified clusters, with clear distinctions in gender, SES and various health-related variables.

Cluster 1 was labelled ‘Higher SES with Elevated Health Risks’ and consisted of individuals with a high SES, predominantly female, who faced notable health-related concerns. The *z*-scores indicated elevated systolic (0.33) and diastolic (0.29) BP, along with relatively high blood glucose (0.12) and cholesterol levels (0.27). This group also reported the highest perceived stress levels (*z*-score = 1.11) among all clusters and had a high BMI score (*z*-score = 1.73), suggesting potential risks associated with weight and stress. The ANOVA results supported the significance of these elevated health indicators, showing high F values (e.g. *F* = 73.838 for systolic BP, *F* = 66.716 for BMI, both with *p* < 0.001), highlighting the distinct risk profile of Cluster 1 despite its higher SES.

Cluster 2 was named ‘Lower SES with Balanced Health Metrics’ and was characterised by predominantly female individuals from a lower socio-economic background, who maintained a relatively healthy profile. Although this cluster showed slightly elevated systolic (0.45) and diastolic (0.57) BP scores, their blood glucose (-0.54) and cholesterol (-0.65) levels were low, indicating a healthier metabolic profile. This cluster also reported relatively low perceived stress (0.29) and a near-average BMI (-0.27). The ANOVA findings further confirmed significant differences in health metrics across clusters, as evidenced by a high F value for cholesterol (*F* = 17.165, *p* < 0.001), suggesting that Cluster 2, despite lower SES, maintained stable health indicators that differentiated it from other clusters with higher metabolic risks.

Cluster 3, labelled ‘Lower SES with Low Blood Pressure and Moderate Health’, included individuals from low SES backgrounds and displayed notably low systolic and diastolic BP scores (both -0.86), indicating reduced BP. Blood glucose (-0.18) and cholesterol (0.06) were moderate in this cluster, and perceived stress levels were low (-0.17), along with a low BMI score (-0.37). These results suggested that Cluster 3, while of low SES, had a relatively stable and balanced health profile with low cardiovascular risks. The ANOVA analysis highlighted significant between-cluster differences in systolic and diastolic BP, affirming that Cluster 3’s low BP values were distinctive and statistically significant (*F* = 73.838 for systolic, *F* = 74.313 for diastolic, both with *p* < 0.001).

Cluster 4, ‘Lower SES with Metabolic Health Concerns’, was also composed of individuals from a lower SES and predominantly female. This cluster had the highest systolic (0.69) and diastolic (0.60) BP scores among all clusters, along with high blood glucose (0.80) and cholesterol (0.45) levels, which pointed to potential metabolic health issues. Despite this, Cluster 4 showed low perceived stress (-0.55) and a BMI close to the average (-0.04). The ANOVA results confirmed significant differences across clusters for all metabolic indicators, with particularly high F values for blood glucose (*F* = 27.776, *p* < 0.001) and BP, underscoring the elevated metabolic risks that uniquely characterised Cluster 4.

## Discussion

The current study aimed to assess the prevalence of NCD risk factors and their association with gender, SES, and perceived stress among adolescents in the Eastern Cape province, South Africa. The study demonstrated a significant difference in the accumulation of NCD risk factors between male and female participants. Females exhibited a notably higher risk of NCDs, with 80.2% classified as overweight or obese, compared to only 19.8% of males. These results are consistent with previous research indicating that adolescent girls in South Africa are more likely to experience obesity and related metabolic conditions than boys (Draper et al. 2019; Otitoola et al. 2021). These disparities could be attributed to socio-cultural factors that influence dietary patterns and physical activity among adolescent girls, particularly in disadvantaged areas. In addition, a larger proportion of females is classified as having elevated cholesterol levels and BP compared to males, further underscoring their greater metabolic burden.

A significant association was observed between SES and perceived stress, with participants from more affluent schools (quintiles 4–5) reporting higher levels of stress compared to their counterparts from disadvantaged schools (quintiles 1–3). These results are somewhat surprising, as adolescents from lower SES backgrounds are typically assumed to experience higher stress because of economic hardships and a lack of resources (Stormacq et al. 2019). However, it is possible that adolescents in wealthier schools face greater academic pressure, social expectations and competitive environments, all of which may contribute to elevated stress levels. This pattern has been observed in other studies, where affluent adolescents reported higher psychological stress despite their better material circumstances, making it difficult for these children to cope with stress in healthy ways (Patalay & Fitzsimons 2018; Van Loon et al. 2020).

Despite the higher stress levels reported by adolescents from wealthier schools, they were less likely to present with multiple metabolic risk factors compared to those from disadvantaged schools. This suggests that the physiological impacts of stress may be modulated by other factors, such as access to healthier food options, better healthcare and opportunities for physical activity in wealthier environments.

Gender differences in perceived stress were also evident, with females reporting significantly higher levels of stress than males. This aligns with existing literature indicating that adolescent girls are more vulnerable to psychological stress because of factors such as body image concerns, social pressures and hormonal changes during adolescence (Reilly & Kelly 2011). The higher levels of stress reported by females in this study may also be linked to their greater burden of metabolic risk factors, as previous research has shown that obesity and metabolic dysregulation can exacerbate stress responses (Carballo et al. 2020; Lindholdt et al. 2022).

The correlation between BMI and perceived stress further supports this connection, suggesting that adolescents with higher BMI are more likely to experience elevated stress levels. This relationship could reflect the psychological impact of being overweight, which may contribute to lower self-esteem and increased social anxiety, particularly among females.

The study also identified significant correlations between BMI, BP and blood glucose levels, with higher BMI being associated with increased systolic BP and blood glucose levels. These results are consistent with the relationship between obesity and hypertension, as excess body fat contributes to increased vascular resistance and metabolic dysfunction (Gooding et al. 2020). The positive correlation between blood glucose and systolic BP suggests that adolescents with elevated glucose levels may also be at higher risk for developing hypertension, a key risk factor for cardiovascular diseases. Similarly, a study by Cheung et al. (2017) found that adolescents with higher blood glucose levels, even within the normal range, showed a greater likelihood of elevated BP later in life.

Although perceived stress was significantly correlated with BMI, it was not directly associated with other metabolic risk factors such as blood glucose or cholesterol levels. This may indicate that while stress influences certain physiological outcomes, such as weight gain, it does not have a uniform impact on all metabolic processes. In addition, stress is linked to psychopathology, including disordered eating, independent of body mass in women across the BMI spectrum (Monyeki et al. 2012; Kivimäki, Bartolomucci & Kawachi 2023). Further research is needed to explore how stress affects different aspects of metabolic health in adolescents.

The cluster analysis revealed important insights into the complex interplay of SES, perceived stress and health metrics, all of which contribute to the risk of NCDs. The results indicated clear variations in the health profiles of participants, with SES playing a significant role in shaping both the physical and mental health of individuals. While higher SES was expected to confer better health outcomes, the findings highlighted that elevated stress and unhealthy lifestyle factors, such as high BMI and BP, could exacerbate NCD risk, even in socio-economically advantaged groups. This aligns with previous studies that have shown that, despite the protective effects of higher SES, stress and lifestyle factors such as diet and physical inactivity can contribute to adverse health outcomes (Mtintsilana et al. 2023; Stringhini et al. 2017).

The analysis also indicated that SES differences did not always correlate directly with better health outcomes. For instance, individuals from lower SES backgrounds exhibited a range of health profiles, some of which showed metabolic concerns such as elevated blood glucose and cholesterol, while others maintained balanced health metrics despite their socio-economic challenges. This finding is consistent with prior research demonstrating that low SES individuals are at heightened risk for metabolic conditions such as diabetes and cardiovascular diseases because of factors such as limited access to healthcare, poor diet and stress (Braveman et al. 2010; Galea et al. 2011; Kechter et al. 2019). However, the variability in health outcomes across the lower SES groups suggests that other factors, such as resilience or access to community resources, might buffer against these risks.

Perceived stress emerged as a crucial factor across all clusters, with high stress levels consistently associated with poor health outcomes. This is in line with research that suggests stress, particularly chronic stress, is a major risk factor for NCDs, contributing to conditions such as hypertension, obesity and metabolic syndrome (Matthews & Gallo 2011; Sapolsky 2004). Interestingly, females across all SES groups reported higher perceived stress and BMI, both of which are known contributors to NCDs, particularly heart disease, diabetes and obesity. This finding underscores the importance of considering gender when designing health interventions, as stress and weight-related health issues disproportionately affect women (Lantz et al. 1998). Furthermore, studies have shown that high stress can lead to the development of metabolic syndrome, which includes abdominal obesity, hypertension and dyslipidaemia – key risk factors for cardiovascular disease (Matthews & Gallo 2011; Letswalo et al. 2021; Kuciene & Dulskiene 2021).

These findings underscore the urgent need for tailored public health interventions that account for socio-economic and gender-specific factors influencing health outcomes. Given the intricate links between SES, stress and metabolic health, interventions should go beyond improving healthcare access and healthy lifestyle promotion. They should also address the socio-economic and psychological stressors that disproportionately impact vulnerable populations, particularly adolescent girls. Gender-sensitive approaches, such as stress management programmes and initiatives targeting BMI and metabolic health, are essential for this group.

Addressing socio-economic disparities through policies that improve healthcare access, availability of nutritious food and opportunities for physical activity can help mitigate health risks associated with lower SES (Braveman et al. 2010; Marmot 2005). For adolescents, targeted interventions should consider the unique stressors of different socio-economic settings. Those in wealthier schools may benefit from support in managing academic and social pressures, while those in disadvantaged environments require a focus on the broader social determinants of health. The associations between BMI, BP and glucose levels further emphasise the importance of early screening and intervention for overweight and hypertension, which could be critical for NCD prevention in adolescents and reducing South Africa’s long-term healthcare burden.

### Limitations and future research

First of all, the cross-sectional design limits the ability to establish causality between perceived stress and NCD risk factors. Longitudinal studies are needed to better understand the temporal relationships between these variables. In addition, the study was conducted in only two districts of the Eastern Cape province, which may limit the generalisability of the findings to other regions of South Africa. Future research should explore the mechanisms underlying the relationship between perceived stress and metabolic risk factors, as well as the role of other psychosocial factors, such as social support and resilience, in moderating these relationships. Moreover, larger studies that include a more diverse sample of adolescents from different socio-economic backgrounds and regions would help to confirm the findings and provide a more comprehensive understanding of NCD risk in South Africa.

## Conclusion

This study highlights the complex interplay of SES, stress and gender in shaping NCD risk among South African adolescents, particularly among females and those from disadvantaged backgrounds. These findings indicate the need for public health interventions that address both physical and psychological risk factors, considering both individual and structural influences. Effective strategies should focus on gender-specific needs, stress management and reducing socio-economic disparities to prevent long-term NCD consequences. By tailoring interventions to the unique needs of diverse socio-economic groups and integrating gender-sensitive approaches, it is possible to address underlying health determinants and reduce NCD incidence in at-risk populations.
